# Acupuncture for the treatment of dry eye: Protocol for systematic review and meta-analysis

**DOI:** 10.1097/MD.0000000000021625

**Published:** 2020-08-07

**Authors:** Yongzheng Zhu, Qinyu Zhao, Hongling Jia, Hongxing Zhang, Yongchen Zhang

**Affiliations:** a College of acupuncture and massage; b Department of Acupuncture, The Second Affiliated Hospital of Shandong University of Traditional Chinese Medicine; c Department of Ophtalmology in Chinese medicine, Shandong Provincial Qianfoshan Hospital, Jinan, Shandong, China.

**Keywords:** acupuncture, dry eye, protocol, systematic review

## Abstract

**Background:**

As a common ophthalmic disease, dry eye (DE) may bring several adverse effects on the quality of life for patients. In recent years, Acupuncture (AC) is becoming increasingly popular for treating DE. Thus, we conceived this systematic review aims to evaluate the effectiveness and safety of AC for DE objectively.

**Methods:**

The search results are restricted to randomized controlled trials and human studies. We will establish the inclusion criteria through discussion and design a detailed literature search strategy for each database. Articles are searched from 4 English databases (the Cochrane Library, PubMed, Web of Science, and EMBASE) and 4 Chinese databases (Wangfang, China National Knowledge Infrastructure, Chinese Biomedical Literature Database, and Chinese scientific and technical journals database). The relevant studies published from the date of database inception until January 2020 will be collected. We will also search (International Clinical Trials Registry Platform), PROSPERO, and potential gray literature. Two reviewers independently perform literature screening, information extraction, and assessment of study quality. The outcome measures include primary outcome measures (Schirmer I test and break-up time), secondary outcome measures (Ocular Surface Disease Index, corneal fluorescein staining, and tear osmolarity), and safety outcome measures. Assessment of bias risk and data processing are performed using RevMan 5.3 software ( the Nordic Cochrane Centre, Copenhagen, Denmark).

**Results:**

We will evaluate the curative effect of AC for DE comprehensively based on multiple outcome measures.

**Conclusion:**

This systematic review will provide evidence for the effectiveness and safety of AC in the treatment of DE.

**PROSPERO number:**

CRD42019144790

## 1 Introduction

Dry eye (DE) is a multifactorial disease of the ocular surface,^[1]^ characterized by abnormalities of homeostasis of the tear film.^[2]^ Dry eye is commonly accompanied by ocular discomfort or visual impairment, including eye fatigue, pain perception, foreign body sensation, burning sensation, blurred vision, and decreased vision.^[3]^ Tear hyperosmolarity is the core pathogenesis causing DE disease. Hypertonic tears can injury the ocular surface and promote inflammation, accelerate the corneal, and conjunctival epithelial cells, goblet cells apoptosis, this will lead to the tear film stability further decreased and aggravation of symptoms in DE patients.^[4]^ DE is a common disease with a prevalence of 5%–50%^[5]^, which results in inconvenience to the patients’ daily life^[6–8]^ and reduces the efficiency of working and studying.^[9,10]^ Age, race, Gender, meibomian gland dysfunction, Sjogren syndrome, estrogen replacement therapy, psychological factors, and certain diseases (such as diabetes,^[11]^ autoimmune disease,^[12]^ thyroid disease,^[13]^) are risk factors for DE. Studies showed a positive association between the incidence of DE and age,^[14,15]^ and the incidence rates among female were higher than among male.^[16,17]^ Besides, certain drugs (such as antidepressants, antihistamines), as well as ophthalmic surgery (such as cataract surgery, corneal transplant surgery, refractive surgery) may also induce DE.^[18]^ The application of artificial tears (AT) is the most common therapeutic method for treating DE. However, most eye drops contain preservatives to inhibit the growth of microorganisms. Studies have shown that preservatives in ATs decreased the stabilization of the tear film, and symptoms of DE patients were exacerbated with long-term AT use.^[19]^ Preservative-free tear drops showed a better curative effect in DE, but at a high economic cost.^[20]^ Meanwhile, complementary and alternative therapies received increasing attention. As an important part of Traditional Chinese Medicine, acupuncture (AC) is recognized as a promising treatment for patients with DE. The number of relevant researches has gradually increased in recent years.^[21–23]^

Previous systematic reviews showed that AC is more effective than AT for patients with DE. However, conclusion due to the low quality of the evidence and marked heterogeneity, the effectiveness of AC for DE was not clear. Furthermore, certain researches implied that AC is more than a placebo. In 1 randomized controlled trials (RCT), 42 DE patients received true and sham AC. After 4 weeks, the results indicated no significant differences in symptoms improvement between the 2 groups.^[24]^ However, another study suggests that true AC was superior to sham AC for reducing DE symptoms.^[25]^ To our knowledge, no meta-analysis has been performed to ascertain whether true AC is superior to placebo AC for DE treatment. Therefore, we perform this systematic review to provide evidence from RCTs to assess the effectiveness and safety of AC for treating DE.

## 2 Method

This systematic review protocol has been registered on PROSPERO. The registration number is CRD42019144790. In our study, the review will follow the Preferred Reporting Items for Systematic Reviews and Meta-Analysis Protocol statement guidelines.^[26]^ As a literature-based study, ethical approval was not required.

### 2.1 Inclusion criteria for this study

#### 2.1.1 Types of studies

Only RCTs of AC treatment for DE will be included without language restrictions. Non-RCTs, animal trails, reviews, case reports, and duplicate publications will be excluded from this study.

#### 2.1.2 Types of participants

Patients (without any ethnic, gender, or age restrictions) diagnosed with keratoconjunctivitis sicca or DE will be included. The reference for diagnostic criteria of DE is as follows: methodologies to diagnose and monitor DE disease suggested by the Diagnostic Methodology Subcommittee of the International DE Workshop (2007).^[27]^ TFOS DEWS II diagnostic methodology report (2017).^[28]^ Expert consensus on diagnosis and treatment of DE formulated by the Chinese Medical Association Ophthalmology Branch (2013).^[29]^

#### 2.1.3 Types of interventions

The treatment group of RCTs which focus on needle AC (for example, body AC, electroacupuncture, scalp AC, or eye-AC) will be included, and non-needle AC (such as laser AC, moxibustion, or acupressure) will be excluded. Control group of RCTs will include ATs treatment, placebo AC, no treatment, or other active intervention. There is no limitation on acupoint selection and AC manipulation in this review.

#### 2.1.4 Types of outcome measures

##### 2.1.4.1 Primary outcomes

The primary outcomes include the Schirmer I test and the tear film break-up time. Schirmer I test is commonly used for the measurement of tear secretion volume.^[30]^ Break-up time is an objective index, reflecting the stability of tear film.^[31]^

##### 2.1.4.2 Secondary outcomes

The secondary outcomes include Ocular Surface Disease Index,^[32]^ corneal fluorescein staining, and tear osmolarity.^[33,34]^ Ocular Surface Disease Index Questionnaire is used to evaluate subjective symptoms of DE. Corneal fluorescein staining is used to visualize corneal injuries. Tear osmolarity is an important indicator used to evaluate the severity of DE.

##### 2.1.4.3 Safety outcomes

The safety outcomes will be presented by the severity and incidence rate of adverse reactions.

### 2.2 Data sources

#### 2.2.1 Electronic searches

We will search 4 English databases, and 4 Chinese databases for relevant RCTs published up to January 2020, including PubMed, The Cochrane Library, EMBASE, Web of Science, Chinese Biomedicine Literature Database, China National Knowledge Infrastructure, Wanfang database, and China Science and Technology Journal Database. The retrieval strategy of PubMed has been detailed in Table 1, which was adapted for use in other databases.

**Table 1:**
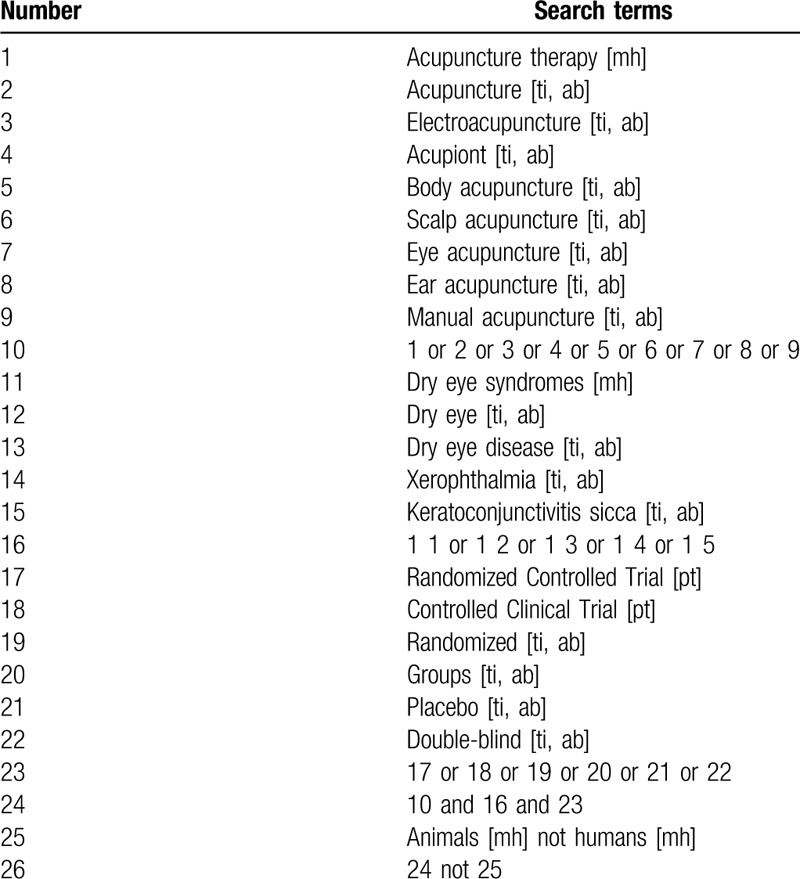
Search strategy used in PubMed database.

#### 2.2.2 Searching other resources

We will search WHO International Clinical Trials Registry Platform, The US National Institutes of Health register, Chinese Clinical Trial Registry, relevant conference proceedings, or grey literature for any potential studies.

### 2.3 Data collection and analysis

#### 2.3.1 Selection of studies

**Figure 1:**
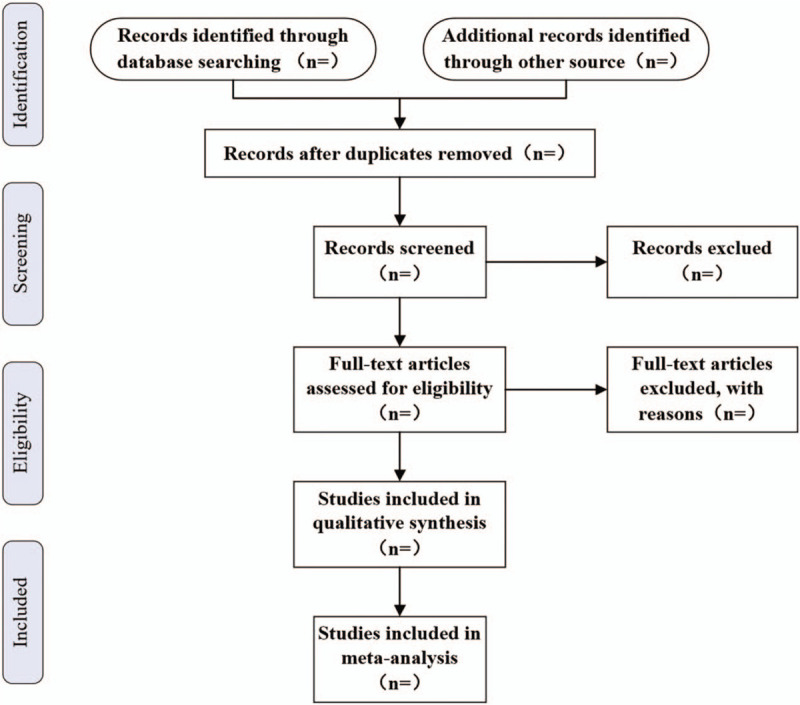
The PRISMA flow chart. PRISMA = Preferred Reporting Items for Systematic Reviews and Meta-Analyses.

The retrieved searches will be imported into EndNote X8 software (Thomson Reuters, New York, America) for literature management and duplicate elimination. Two independent reviewers (YZZ and QYZ) will screen retrieved articles for eligibility initially according to title and abstract, and then check results with each other. When disagreements arise, the final decision will be determined by discussion with a third reviewer (HXZ). Full-text reading will be performed by them if necessary. The selection procedure in our study is detailed in the Preferred Reporting Items for Systematic Reviews and Meta-Analysis Protocol flow chart (Fig. 1).

#### 2.3.2 Data extraction

Two researchers (YZZ and QYZ) will be responsible for data extraction with a predesigned form, follow by cross-checking. Any divergence of views will be resolved by consulting a third investigator (HLJ). The contents in the form are listed as follows: title, author, trial design, numbers of participants, diagnostic criteria, types of interventions, course of treatment, follow-up time, outcome measures, and side effects.

#### 2.3.3 Risk of bias assessment

Two reviewers (YZZ and QYZ) will evaluate the methodological quality of the included literature with the Cochrane Risk of Bias Tools recommended by the Cochrane Handbook.^[35]^ A third author (YCZ) will make the final decision in case of disagreement. The assessment tool contains 7 domains: random sequence generation, allocation concealment, blinding of participants and personnel, blinding of outcome assessment, incomplete outcome data, selective reporting, and other bias. Each domain will be evaluated as high risk, unclear risk, or low risk.

#### 2.3.4 Measures of treatment effect

The dichotomous variable will be shown by relative risk, and the continuous variable will be shown by weighted mean difference or standardized mean difference. Interval estimates will all be based on 95% confidence intervals.

#### 2.3.5 Dealing with missing data

We will try to resort to the primary authors via phone or E-mail for any missing or vague data. If we fail to receive responses, the corresponding articles will be waived due to unavailable data.

#### 2.3.6 Assessment of heterogeneity

We will assess the heterogeneity according to I^2^ statistical values.^[36,37]^ The thresholds of I^2^ are interpreted in the Cochrane Handbook as follows: 0%–40%: might not be important, 30%–60%: may represent moderate heterogeneity, 50%–90%: may represent substantial heterogeneity, and 75%–100%: considerable heterogeneity. Meta-regression and subgroup analysis will be conducted to detect the factors of heterogeneity between the outcomes of studies in case of substantial or considerable heterogeneity.

#### 2.3.7 Assessment of reporting biases

Funnel plots will be utilized to evaluate the reporting biases if a considerable number of RCTs (≥10) are included in the meta-analysis.

#### 2.3.8 Data synthesis

We will analyze the data of included studies with Review Manager V.5.3 software (the Nordic Cochrane Centre, Copenhagen, Denmark). The choice between a fixed-effect and a random-effects model will be made based on I^2^ values. We will perform data synthesis with a fixed-effect model if there is insignificant heterogeneity (I^2^≤50) between studies; otherwise, a random-effect model will be applied. Outcomes which are not appropriate for quantitative synthesis will be reported in narrative form. The results will also be presented in the form of forest plots.

#### 2.4.9 Subgroup analysis

We plan to perform a subgroup analysis to explore reasons for heterogeneity between the studies. Grouping factors are listed as follows:

1. Types of AC.
2. Types of ATs used in treatment.
3. The measuring time of outcomes.

#### 2.4.10 Quality of the evidence

We will evaluate the systematic review findings and quality of evidence through the Grading of Recommendations Assessment, Development, and Evaluation approach.^[38]^ The strength of evidence will be described with 4 categories: very low, low, moderate, or high.

## 3 Discussions

The DE incidence increased gradually due to environmental pollution and the overuse of visual display terminal in recent years.^[39,40]^ AC treatment for DE attracted increasing attention of scholars, considering its benefit of simple operation, low price, and fewer side effects. Although the mechanism of AC for DE is still not completely clarified, an experimental study has revealed that AC could reduce the concentration of proinflammatory cytokines (TNF-α, IL-4, IL-12) in tears.^[41]^ Other studies have shown that AC could alter the content of some proteins in tears and promote lacrimal gland secretion.^[42,43]^

Recommendations for the clinical trial design of DE have been given by the TFOS DEWS Clinical Trial Subcommittee.^[44]^ This means more high-quality clinical studies will be published. Therefore, we proposed this systematic review aims to provide reliable evidence for the effectiveness and safety of AC therapy for DE. We hope that the results could help clinicians and patients to make a better choice in treatment selection when facing DE.

## Author contributions

**Investigation:** Yongzheng Zhu, Yongchen Zhang.

**Methodology:** Yongzheng Zhu, Qingyu Zhao, Hongxing Zhang.

**Resources:** Yongzheng Zhu, Qingyu Zhao, Hongling Jia.

**Supervision:** Yongchen Zhang.

**Writing – original draft:** Yongzheng Zhu.

**Writing – review & editing:** Yongchen Zhang.
